# Biomechanical analysis using finite element analysis of orbital floor fractures reproduced in a realistic experimental environment with an anatomical model

**DOI:** 10.3389/fbioe.2024.1354944

**Published:** 2024-05-07

**Authors:** Changryul Claud Yi, Jaehoon Kim, Jaebong Jung, Deoksang Jo, Ji Hoon Kim

**Affiliations:** ^1^ Department of Plastic and Reconstructive Surgery, Pusan National University School of Medicine, Busan, Republic of Korea; ^2^ Biomedical Research Institute, Pusan National University Hospital, Busan, Republic of Korea; ^3^ School of Mechanical Engineering, Pusan National University, Busan, Republic of Korea

**Keywords:** finite element analysis, tomography, X-ray computed, orbital fractures, orbit

## Abstract

**Introduction:** In this study, we attempted to demonstrate the actual process of orbital floor fracture visually and computationally in anatomically reconstructed structures and to investigate them using finite element analysis.

**Methods:** A finite element model of the skull and cervical vertebrae was reconstructed from computed tomography data, and an eyeball surrounded by extraocular adipose was modeled in the orbital cavity. Three-dimensional volume mesh was generated using 173,894 of the 4-node hexahedral solid elements.

**Results:** For the cases where the impactor hit the infraorbital foramen, buckling occurred at the orbital bone as a result of the compressive force, and the von Mises stress exceeded 150 MPa. The range of stress components included inferior orbital rim and orbital floor. For the cases where the impactor hit the eyeball first, the orbital bone experienced less stress and the range of stress components limited in orbital floor. The critical speeds for blowout fracture were 4 m/s and 6 m/s for buckling and hydraulic mechanism.

**Conclusion:** Each mechanism has its own fracture inducing energy and its transmission process, type of force causing the fracture, and fracture pattern. It is possible to determine the mechanism of the fracture based on whether an orbital rim fracture is present.

## 1 Introduction

Blowout fractures are one of the most common facial bone fractures in trauma (Nagasao et al., 2010a). Two major theories explain the mechanism of blowout fractures—the buckling mechanism and the hydraulic mechanism. The buckling mechanism assumes that the impact on the infraorbital rim transmits directly to the orbital floor, resulting in a fracture of the thinnest part (Fujino, 1974; Fujino et al., 1974; Tajima et al., 1974; He et al., 2007). The hydraulic mechanism assumes that the impact on the orbit spreads through the orbital contents and blows out the fragile portion of the orbital wall (Rhee et al., 2002).

The buckling mechanism was first proposed by Le Fort through an experimental study applying direct blows on cadavers (Le Fort, 1972). Pfeiffer was the first to propose a hydraulic mechanism by compression of intraorbital soft tissue to fracture the thinnest parts of the orbital wall (Pfeiffer, 1943). Because of the clinical importance of blowout fractures, there have been significant experimental studies that have investigated the main mechanism for blowout fractures (Jones and Evans, 1967; Green et al., 1990; Waterhouse et al., 1999; Ahmad et al., 2003; Ahmad et al., 2006).

Despite these efforts, the mechanism of blowout fracture remains controversial in the literature (Whiting et al., 1988; Ahmad et al., 2006). This is due to the difficulty of reproducing the actual fracture. Computer simulation with finite element analysis is one of the methodologies to be considered for reproducing the process of fracture. Finite element analysis could provide a highly controlled experimental environment and results as numerical data. Analysis of facial bone fracture with finite element analysis has been increasingly used in recent studies (Huempfner-Hierl et al., 2015; Nagasao et al., 2018), and the possible correlation between the actual fracture and finite element analysis has been verified (Szwedowski et al., 2010).

In this study, we attempted to demonstrate the actual process of these mechanisms visually and computationally in anatomically reconstructed structures and to investigate them using finite element analysis in various experimental environment. And through this, the two mechanisms are compared, and the differences are investigated.

## 2 Materials and methods

Our institutional review board approved this study (No. 2012-003-097). Finite element simulations were performed to evaluate the deformation and stress evolution in the orbital bone during impact. The commercial finite element software Abaqus/Explicit (Vélizy-Villacoublay, Île-de-France, France) was used.

**FIGURE 1:**
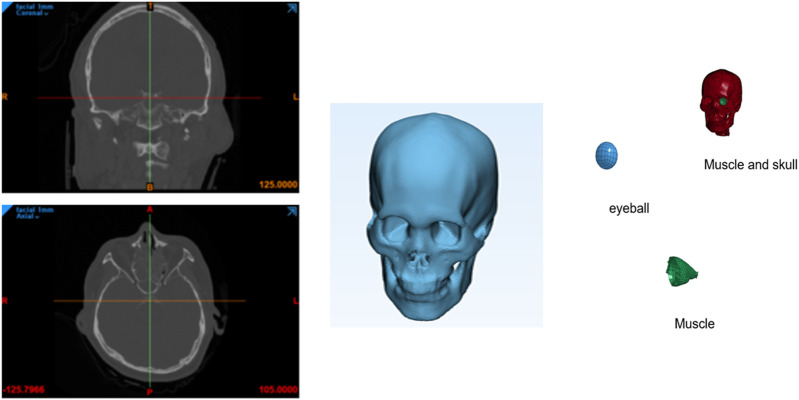
Process of finite element analysis modeling.

A finite element model of the skull and cervical vertebrae was reconstructed from a computed tomography (CT) scan data of the head and computed with MIMICS software (Materialise), as shown in Figure 1. First, the bone region was segmented from the CT data by applying the threshold value of 226. Then, irrelevant regions created by noise were removed from the region of interest. And then three-dimensional volume mesh was generated using the 4-node tetrahedral solid elements (C3D4). In this work, 3D solid elements were used instead of shell elements that have been used in other works. Solid elements can express surface shape and thickness variation better and are expected to provide more accurate results than shell elements.

The thickness of the orbital bone generated by this procedure was much larger than the actual thickness because of the resolution of the CT data. Therefore, the orbital bone thickness was reduced from 1.69 mm to 0.26 mm manually using the LS-PrePost (Livermore Software Technology Corp.). Also, the finite element model was remeshed to increase the number of elements in the orbital bone in the thickness direction so that there are at least 3 layers of elements. This procedure is important to accurately describe the bending behavior of the orbital bone during impact. The total number of elements were 173,894. The eyeball has a complex structure, but in this paper, the eyeball was modeled as a simple homogeneous structure. In the case of orbital fracture, the cornea and iris were ignored.

A spherical eyeball surrounded by extraocular adipose was modeled in the orbital cavity. The impactor of an earlier work (Takizawa et al., 1988), was adopted: a step cylinder was used with a mass of 412 g and a diameter of 20 mm on the impacting side. Linear elasticity was used for the eyeball, adipose, bone, and impactor. The Young’s moduli and Poisson’s ratios of the materials are listed in Table 1. The material properties of the contents were also entered appropriately for each component (Uchio et al., 1999; Power, 2001).

**TABLE 1:** Material parameters.

Material	Young’s modulus (MPa)	Poisson’s ratio	Density (kg/m^3^)
Bone	11,483 (Nagasao et al., 2010b)	0.338	1,728
Eyeball	4 (Hugar and Ivanisevic, 2013)	0.445	1,050
Adipose	1.2 (Comley and Fleck, 2010)	0.49	900
Impactor	100,000 (Takizawa et al., 1988)	0.37	8,400

**FIGURE 2:**
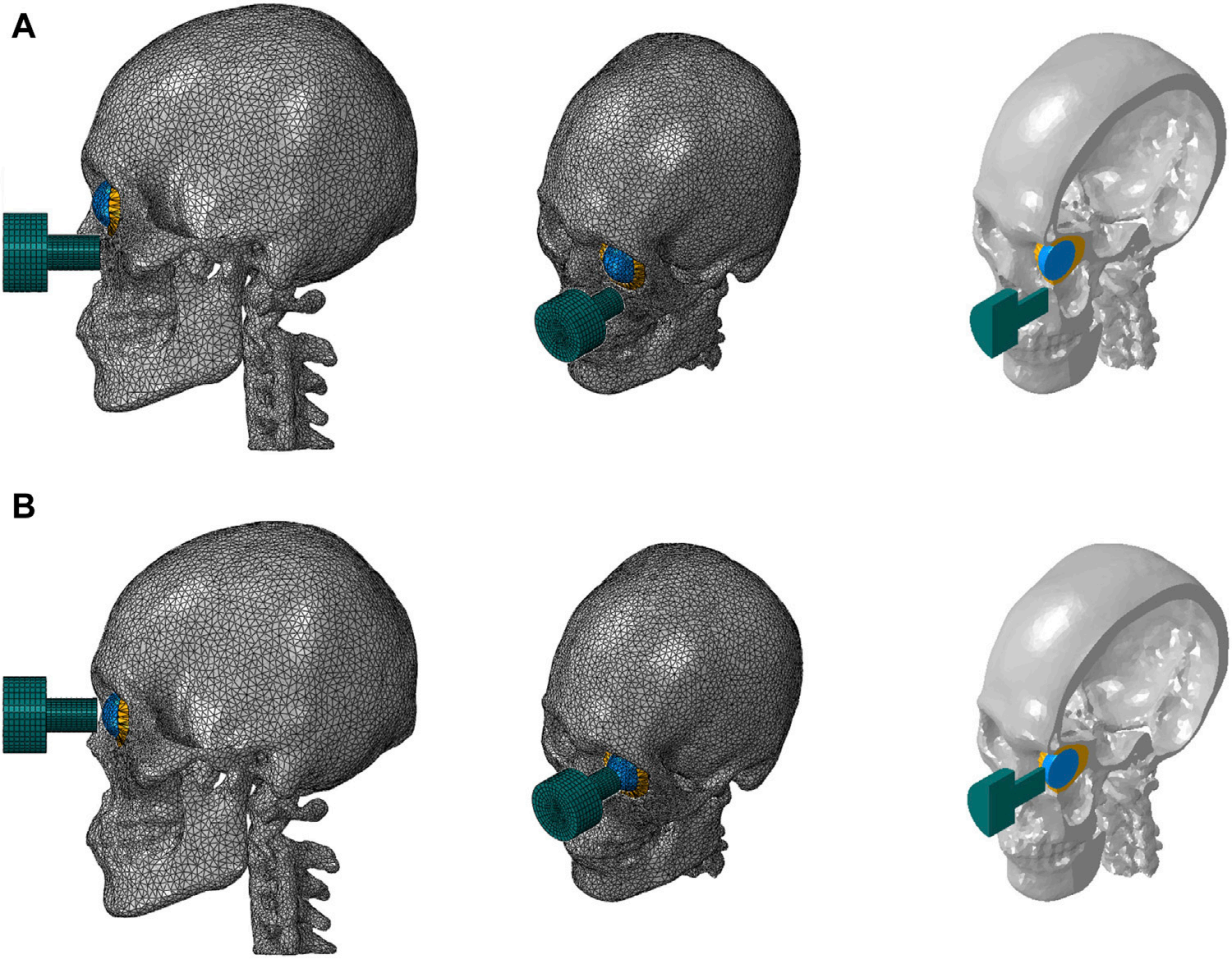
Finite element models of the skull and cervical vertebrae of the two cases where the impactor hit the bone first **(A)** and the eyeball first **(B)**.

**FIGURE 3:**
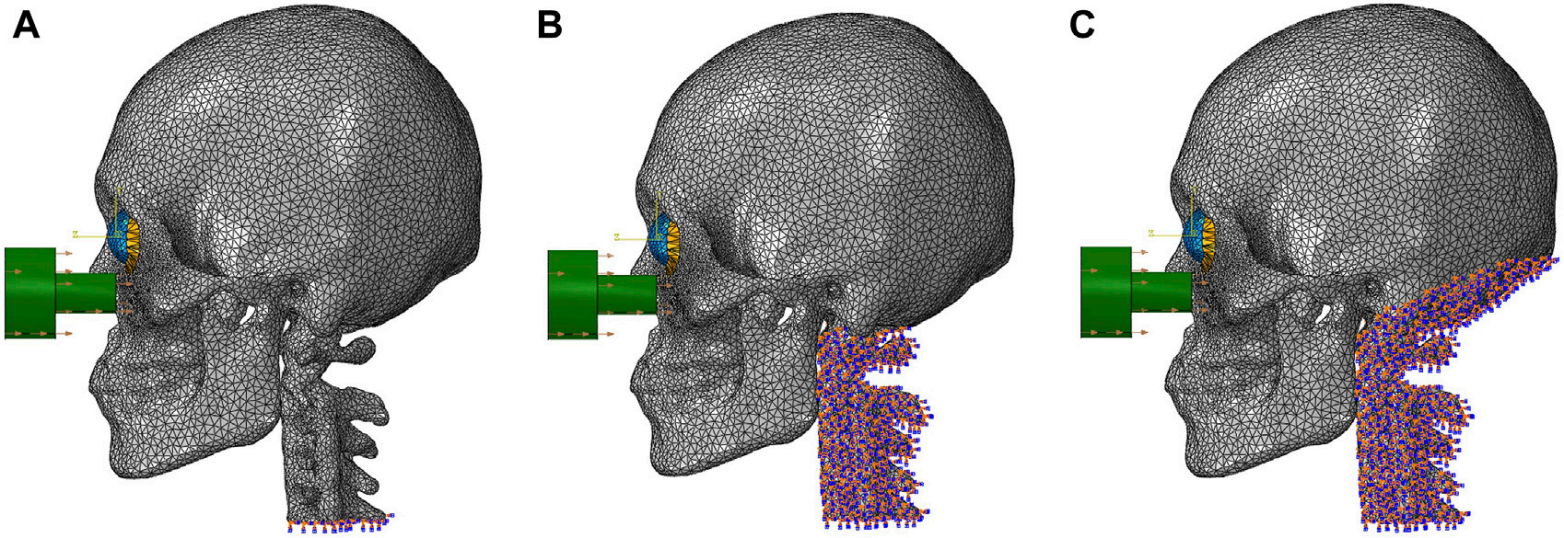
The locations of the fixed boundary conditions: **(A)** the bottom of the cervical vertebrae, **(B)** the entire cervical vertebrae, and **(C)** the occipital and the entire cervical vertebrae.

Two cases were considered. The impactor hit the infraorbital foramen for buckling mechanism and the eyeball first for hydraulic mechanism, as shown in Figure 2. Finite element analysis was performed for a total of 5 cases with impactor speeds of 2, 3, 4, 5, and 6 m/s. To ensure accuracy of analysis, mass scaling was not used. In addition, to evaluate the effect of boundary conditions of the impact simulation, three cases of boundary conditions were imposed where the bottom of the cervical vertebrae was fixed, all cervical vertebrae were fixed, and the occipital and all cervical vertebrae were fixed during the simulation, as shown in Figure 3. For all fixations, the three displacement degrees of freedom was set to vanish: u_1_ = u_2_ = u_3_ = 0. Table 2 summarizes the simulation cases.

**TABLE 2:** Simulation cases.

Cases	Impact position	Fixed boundary condition
A-1	Infraorbital foramen	Bottom of cervical vertebrae
A-2	Infraorbital foramen	Entire cervical vertebrae
A-3	Infraorbital foramen	Occipital and entire cervical vertebrae
B-1	Eyeball	Bottom of cervical vertebrae
B-2	Eyeball	Entire cervical vertebrae
B-3	Eyeball	Occipital and entire cervical vertebrae

## 3 Results

**FIGURE 4:**
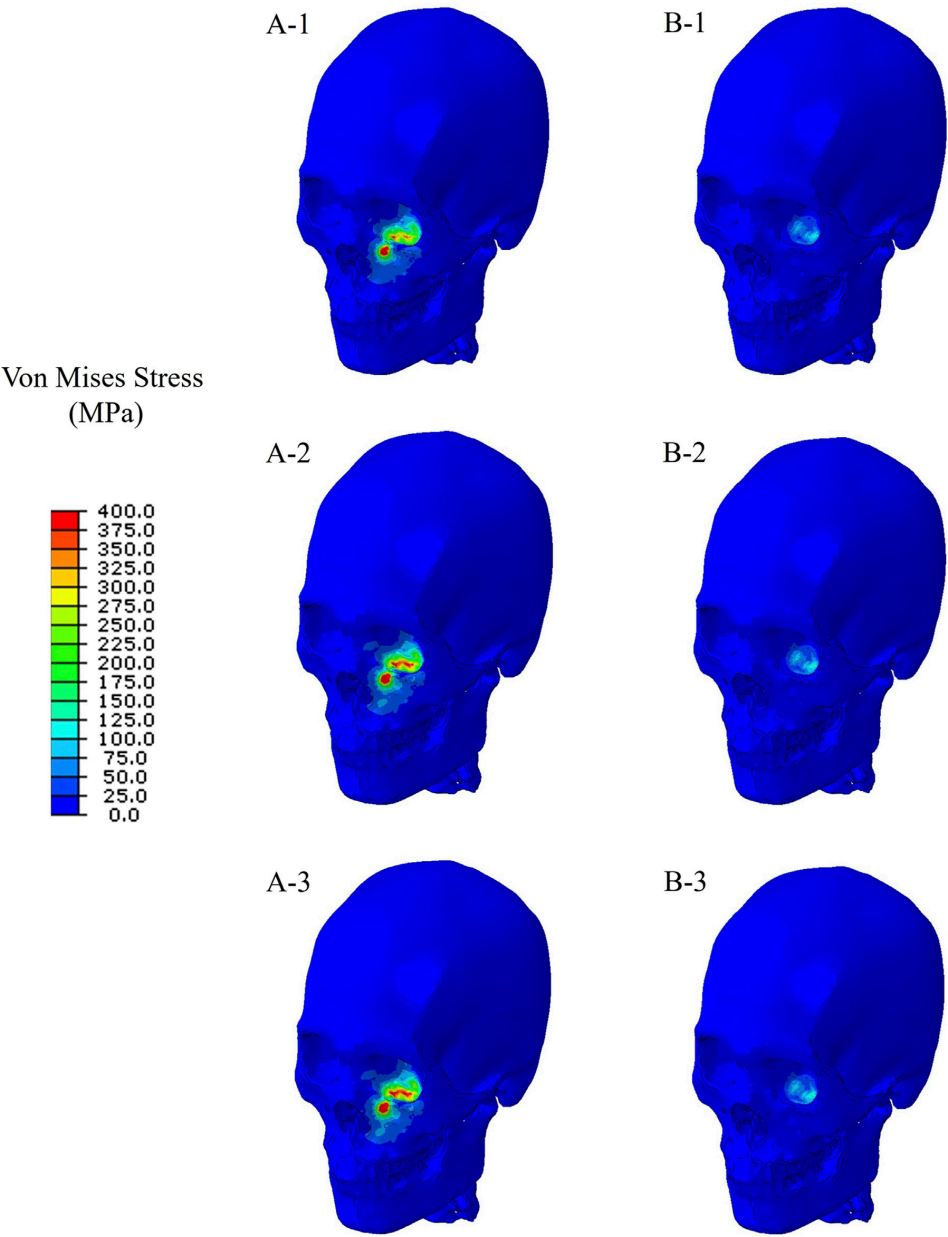
Stress distribution when the stress at the orbital bone is at maximum for the six cases. **(A-1)** The bone impacted, the bottom of the cervical vertebrae fixed. **(A-2)** The bone impacted, the entire cervical vertebrae fixed. **(A-3)** The bone impacted, the occipital and entire cervical vertebrae fixed. **(B-1)** The eyeball impacted, the bottom of the cervical vertebrae fixed. **(B-2)** The eyeball impacted, the entire cervical vertebrae fixed. **(B-3)** The eyeball impacted, the occipital and entire cervical vertebrae fixed.

**FIGURE 5:**
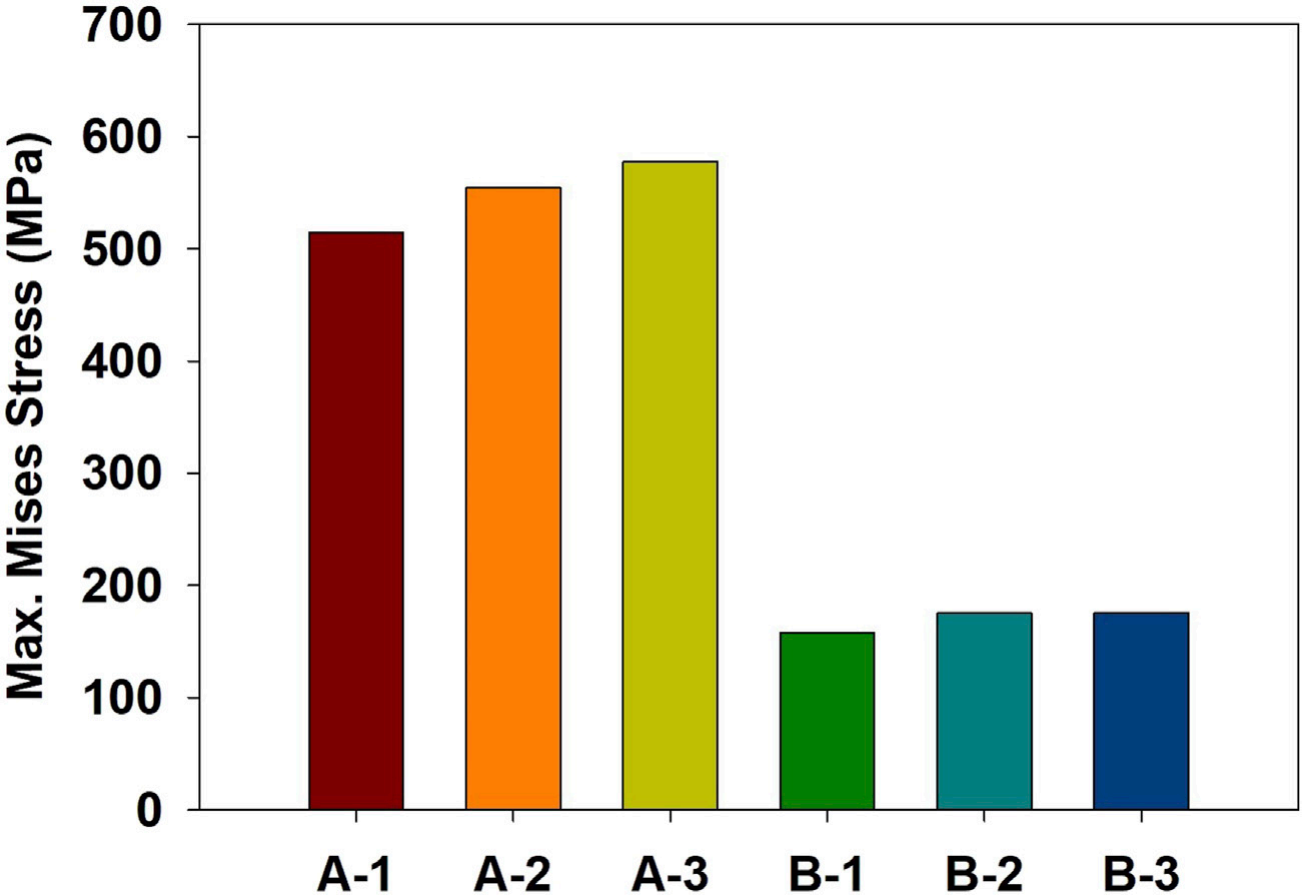
Maximum von Mises stress of the six cases.

Finite element simulations showed that the maximum stress occurred at the orbital bone in all cases. Figure 4 compares the stress distribution when the stress at the orbital bone is at maximum, and Figure 5 shows the maximum stresses of the six cases.

**FIGURE 6:**
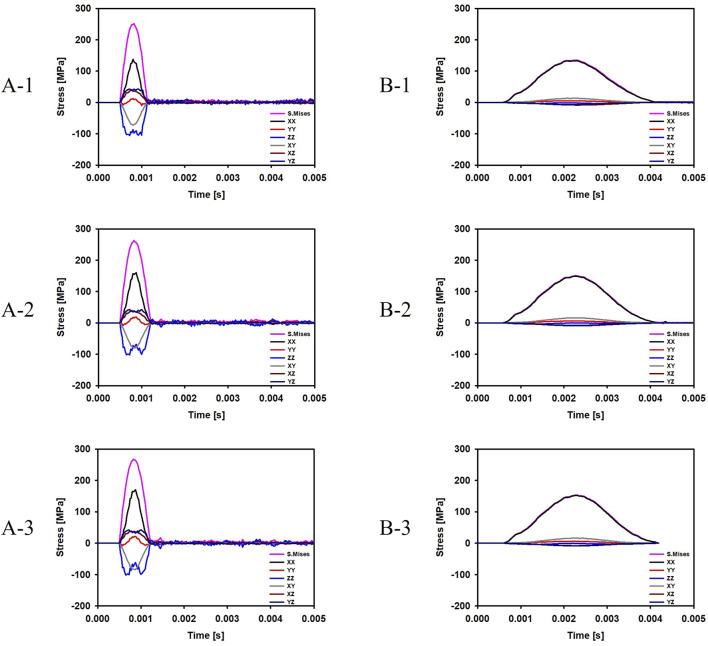
Evolution of the stress components at the top and bottom surfaces of the orbital bone for the six cases. **(A-1)** The bone impacted, the bottom of the cervical vertebrae fixed. **(A-2)** The bone impacted, the entire cervical vertebrae fixed. **(A-3)** The bone impacted, the occipital and entire cervical vertebrae fixed. **(B-1)** The eyeball impacted, the bottom of the cervical vertebrae fixed. **(B-2)** The eyeball impacted, the entire cervical vertebrae fixed. **(B-3)** The eyeball impacted, the occipital and entire cervical vertebrae fixed.

**FIGURE 7:**
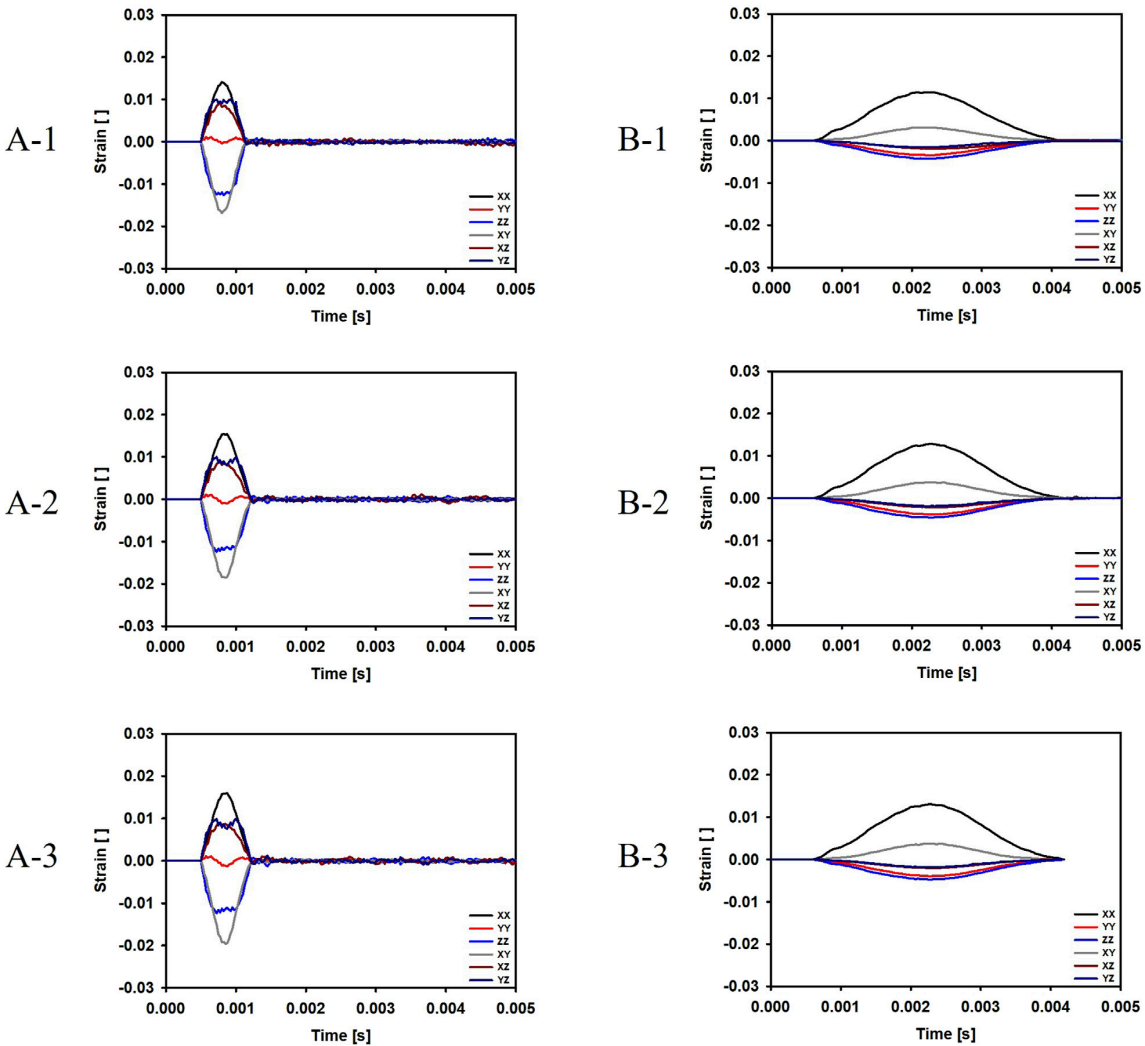
Evolution of the strain components at the top and bottom surfaces of the orbital bone for the six cases. **(A-1)** The bone impacted, the bottom of the cervical vertebrae fixed. **(A-2)** The bone impacted, the entire cervical vertebrae fixed. **(A-3)** The bone impacted, the occipital and entire cervical vertebrae fixed. **(B-1)** The eyeball impacted, the bottom of the cervical vertebrae fixed. **(B-2)** The eyeball impacted, the entire cervical vertebrae fixed. **(B-3)** The eyeball impacted, the occipital and entire cervical vertebrae fixed.

For the cases where the impactor hit the infraorbital foramen (Cases A-1, A-2, A-3), buckling occurred at the orbital bone as a result of the compressive force, and the von Mises stress exceeded 150 MPa which is known as the yield strength of the skull (Nagasao et al., 2010b), as shown in Figure 6. Figure 7 shows the occurrence of a strain of approximately 0.015. The range of the stress components was from the inferior orbital rim to the orbital floor, as shown in Figure 4.

**FIGURE 8:**
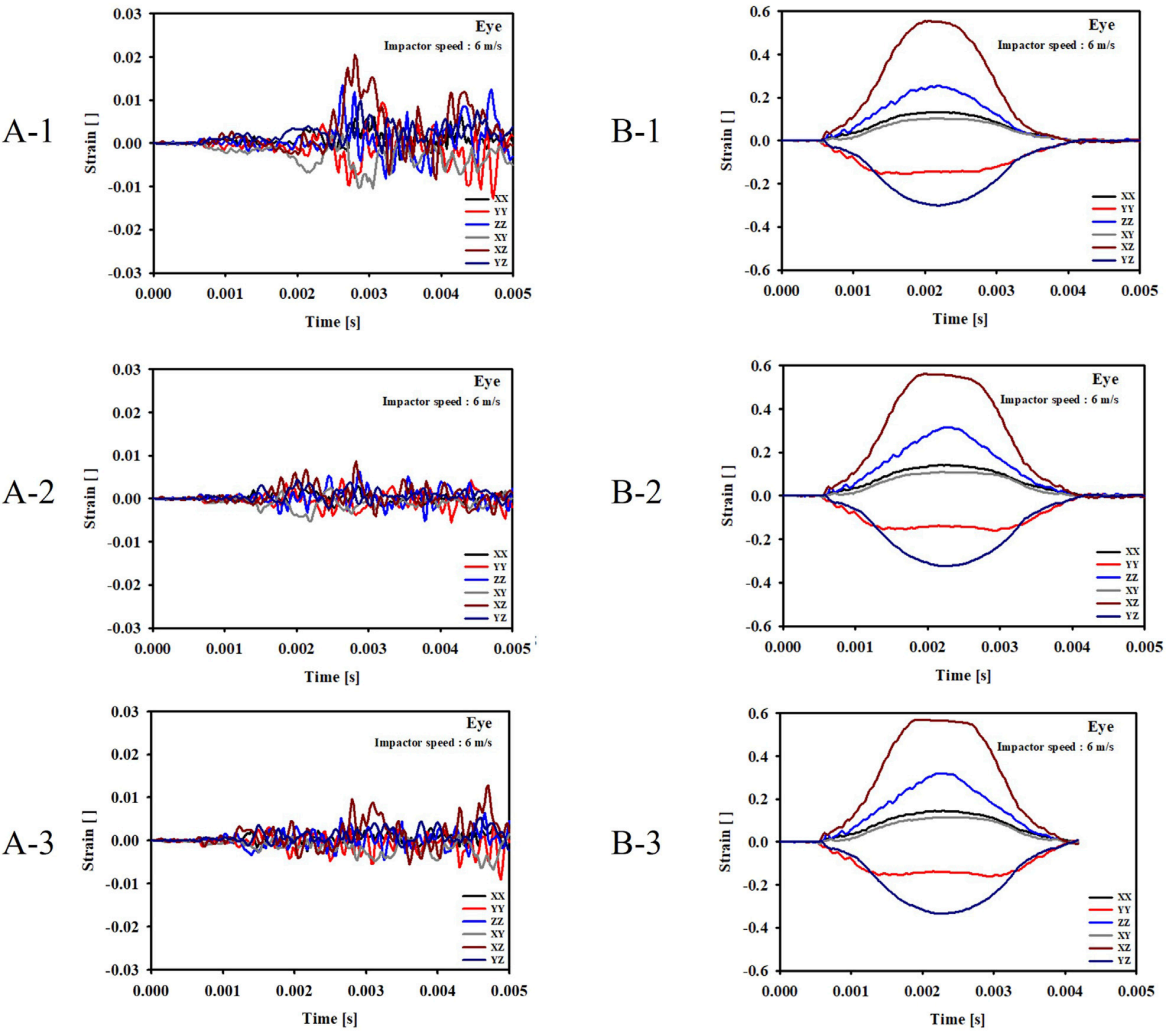
Evolution of the strain components at the eyeball for the six cases. **(A-1)** The bone impacted, the bottom of the cervical vertebrae fixed. **(A-2)** The bone impacted, the entire cervical vertebrae fixed. **(A-3)** The bone impacted, the occipital and entire cervical vertebrae fixed. **(B-1)** The eyeball impacted, the bottom of the cervical vertebrae fixed. **(B-2)** The eyeball impacted, the entire cervical vertebrae fixed. **(B-3)** The eyeball impacted, the occipital and entire cervical vertebrae fixed.

For the cases where the impactor hit the eyeball first (Cases B-1, B-2, B-3), most of the momentum was first absorbed by the eyeball and the adipose tissue, and then transferred to the orbital bone. As a result, the impact duration was longer and the orbital bone experienced less stress than the cases where the impactor hit the infraorbital foramen, as shown in Figures 6, 7. Because a large momentum is applied to the eyeball, the strain at the eyeball is much larger for Case B than for Case A, which may result in damage in the eyeball, as shown in Figure 8. The range of the stress components was limited to the orbital floor, as shown in Figure 4.

**FIGURE 9:**
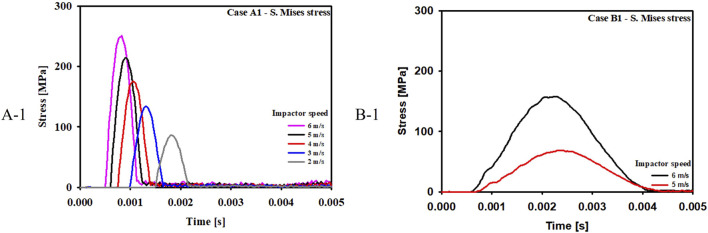
Evolution of the stress components at various impactor speeds for Case A-1 and B-1.

Figure 9 compares the stress at various impactor speeds for Case A-1 and B-1. The critical speeds for blowout fracture were 4 m/s and 6 m/s for Case A-1 and Case B-1, respectively.

In terms of boundary conditions, it was found that for the same impact condition, the maximum stress was the smallest when the bottom of the cervical vertebrae was fixed (Case A-1 and Case B-1). This is attributed to the bending of the cervical vertebrae that absorb the impact energy and increase the impact duration.

## 4 Discussion

In this study, we intend to clearly elucidate the actual process by which two different mechanisms cause orbital floor fractures. We emphasized the implementation of anatomical structures and realistic experimental conditions into the computer model as much as possible for the reliability of the experiment in this study.

CT data from a male in twenties without a history of medical or facial trauma was utilized to build the finite element model of the skull for appropriate simulation. Therefore, the skull model represented an anatomically smooth contour, and realistic bone thickness could be achieved without bias related to post-mortem and senile changes in the facial bone.

The thickness of the orbital floor was also a focus in the implementation of the model. The thickness of the orbital floor was estimated based on the average value from the literature (Jones and Evans, 1967).

Orbital contents are also a very important part in the reproduction of blowout fractures, especially with hydraulic mechanisms. In the past, most studies of blowout fractures using computer simulation have ignored the orbital contents or only included eyeballs (Takizawa et al., 1988; Al-Sukhun et al., 2006; Nagasao et al., 2006; Nagasao et al., 2010a; Nagasao et al., 2010b; Al-sukhun et al., 2012; Schaller et al., 2013). Orbital contents such as orbital fat tissue as well as the eyeball itself were included in this study for the accuracy of the experiment. The shapes of the orbital contents were anatomically realized based on CT data.

The fixation point on the cervical vertebrae was another focus to make this simulation more realistic. From the perspective of a clinician treating actual patients, it is unrealistic to reproduce an orbital fracture by simply impacting a fixed skull in a laboratory situation. In most actual fracture situations, the high-mass trunk part is almost fixed, and the highly mobile cervical spine absorbs a significant portion of the impact applied to the unfixed skull. To reproduce this actual fracture situation more realistically and to check whether there was a difference in fracture pattern depending on the fixation site, simulations were conducted in three different boundary conditions. In the setting where the fixation point is located in the lower cervical spine, the head naturally reclines back after impact and absorbs some of the impact energy in this simulation.

As shown in the results, the maximum stress is the lowest in the boundary condition, which simulates the actual fracture situation as realistically as possible by fixing the lower cervical spine rather than the skull. This suggests a shock-absorbing effect of the unfixed skull and flexible cervical spine. Therefore, in an actual fracture situation, fractures are expected to be induced at significantly higher energy than the fracture-inducing impact derived from previous studies conducted with the skull fixed. Future studies related to head and neck impacts must include consideration of boundary conditions. In addition, this has significant medical implications as it proves that it is important to check whether the head and neck were fixed at the time of injury when examining patients in actual clinical practice.

As a result of this study, two mechanisms, buckling and hydraulic, showed definite, distinguished patterns in this simulation.

First, collision energies were transmitted in different ways. The energy began at the point of impact, transmitted to the thinnest portion of the orbital floor, and then spread to the inferior orbital rim and other parts of the facial bone in the buckling mechanism. In contrast, the energy was focused only on the thinnest portion of the orbital floor except for the orbital rim in the hydraulic mechanism. This suggests that the key factor distinguishing between the two mechanisms of orbital fracture is whether the impact energy targets the orbital rim.

Second, the type of force causing the fracture was different between the two mechanisms. Fracture in the buckling mechanism was affected by the compressive force, which was transmitted in a direction parallel to the orbital floor. However, in the hydraulic mechanism, fracture occurred due to the tensile force, which was developed by the eyeball and the orbital contents in the balloon shape.

Third, there was a definite difference in the critical speeds of impactor, which induced fracture. The critical speed for fracture was 4 m/s in the buckling mechanism and 6 m/s in the hydraulic mechanism. It can be concluded that the buckling mechanism can occur at a much lower impact speed than the hydraulic mechanism because the eyeball absorbs impact energy before transferring it to the orbital bone.

Fourth, these differences made a critical distinction in actual fractures. The fracture area should include the orbital rim as well as the orbital floor in the buckling mechanism, but the fracture area in the hydraulic mechanism was confined to the orbital floor.

This finite element analysis of orbital fracture showed that buckling and hydraulic mechanisms are clearly different mechanisms of orbital fracture, not controversial ones of choice. Each mechanism has its own energy transmission process, type of force causing the fracture, critical speed of impactor and pattern of fracture. The two mechanisms share neither of these points. It is now necessary to end the controversy over which mechanism is the main cause of orbital fracture, to separate the cases of fractures based on mechanism according to clinical patterns and conduct treatment and research separately.

This study established that patients experiencing orbital floor fractures through the buckling mechanism often have concurrent fractures in the inferior orbital rim. In patients suspected of undergoing the buckling mechanism, the possibility of an inferior orbital rim fracture should be kept in mind, and evaluation of the orbital rim fracture should be made before surgery through physical examination or imaging studies. In addition, intraoperative evaluation is necessary to visually check for fractures of the inferior rim that were undetected during the preoperative assessment. In patients with confirmed fractures, the fixation of the orbital rim and zygomaticomaxillary complex should be considered, as well as reconstruction of the orbital fracture. On the other hand, patients affected by the hydraulic mechanism and confirmed to have no fracture on the zygomaticomaxillary complex or inferior orbital rim during surgery would only need to be reconstructed without further fracture correction.

Previous studies on blowout fractures using three-dimensional models based on finite element analysis have used CT-data derived from dry human skulls (Nagasao et al., 2006; Nagasao et al., 2010a; Nagasao et al., 2010b). In the data derived from the studies, post-mortem changes could cause a significant bias. Oversimplification is another drawback in several studies using computer simulations. Owing to technological limitations at the time of the study such as computer processing speed and storage space, the simulation model cannot avoid some part of the simplification. However, the accuracy of finite element analysis depends directly on the accuracy of the relationship between geometry, material properties, and the boundary conditions, which were entered in computer simulations (Al-sukhun et al., 2012). Especially in the analysis of complicated and delicate structures such as the orbit, the simplification severely compromises the reliability of the results. Recently, there has been a finite element analysis study by Foletti et al. (2019) that overcomes these limitations by using an anatomical and sophisticated model. Although it demonstrated the validity of finite element analysis in analyzing the behavior of the human orbit when stress is applied, the data was limited to compare and analyze the two mechanisms of orbital fracture. On the other hand, simulations were performed for various cases by varying the fixation point, impactor speed, and impact position in this study.

The limitations of current technology in modeling should be considered. The resolution of CT scans is limited in the accurate imaging of thin structures such as orbital walls. Currently, CT scans have a slice thickness of approximately 1 mm, an average distance of 0.44 mm, and a resolution of 0.44 mm. As a result, we manually generated the weakest parts of the orbital wall that are most important in blowout fractures because the average thickness of the thinnest parts of the orbital wall is 0.26 mm (Jones and Evans, 1967). The limited number of elements was another limitation. For a more realistic simulation, more elements should be created per unit area. However, the number of elements that can be calculated requires an increased computer processing speed, and the modeling precision is limited by the computer technology at that time.

To the best of our knowledge, this is the most factual finite element analysis for comparing the two mechanisms of orbital floor fractures using anatomically realistic models. This factual finite element analysis of orbital fracture showed that buckling and hydraulic mechanisms clearly differ for orbital floor fractures. Each mechanism has its own fracture inducing energy and its transmission process, type of force causing the fracture, and fracture pattern. We can now end the controversy over which is the main mechanism of orbital fracture. The cases of each mechanism should be classified according to clinical patterns, with or without orbital rim fracture, and separate treatment and research should be conducted. In the buckling mechanism, both pre- and intraoperative evaluations for accompanying inferior orbital rim and zygomaticomaxillary fractures should be made, and the fractures should be corrected, if necessary. On the other hand, if no inferior orbital rim fracture is found, then the fracture is caused by the hydraulic mechanism and only reconstruction of the orbital floor is required.

## Data Availability

The raw data supporting the conclusion of this article will be made available by the authors, without undue reservation.
