# Supplementary figures and images for: Biomechanical analysis using finite element analysis of orbital floor fractures reproduced in a realistic experimental environment with an anatomical model
Source: Front Bioeng Biotechnol. 2024 May 7;12:1354944. doi: 10.3389/fbioe.2024.1354944 (PMC11106499; doi:10.3389/fbioe.2024.1354944)

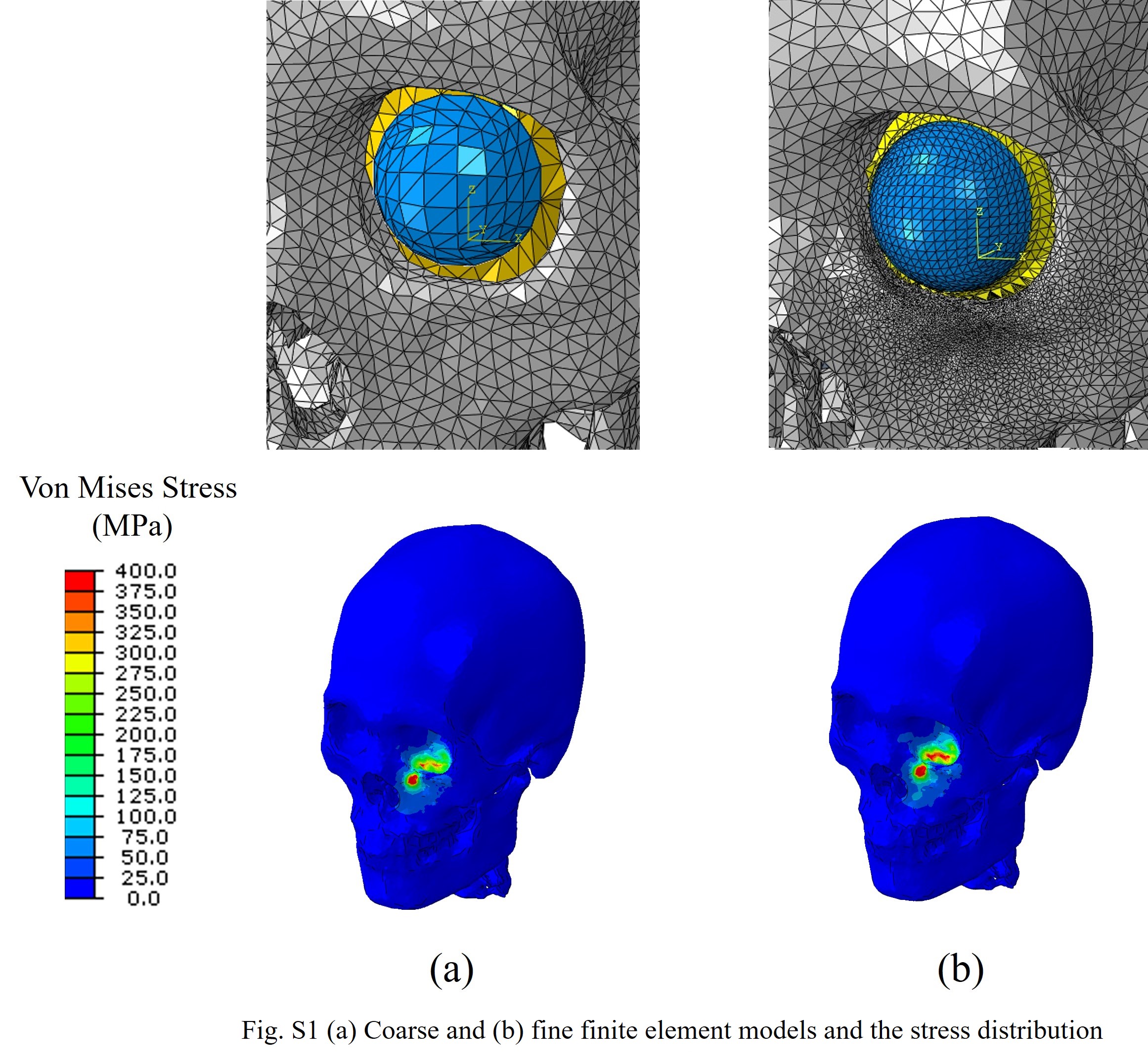

Supplement: Supplementary file 3 [file Image2.JPEG]

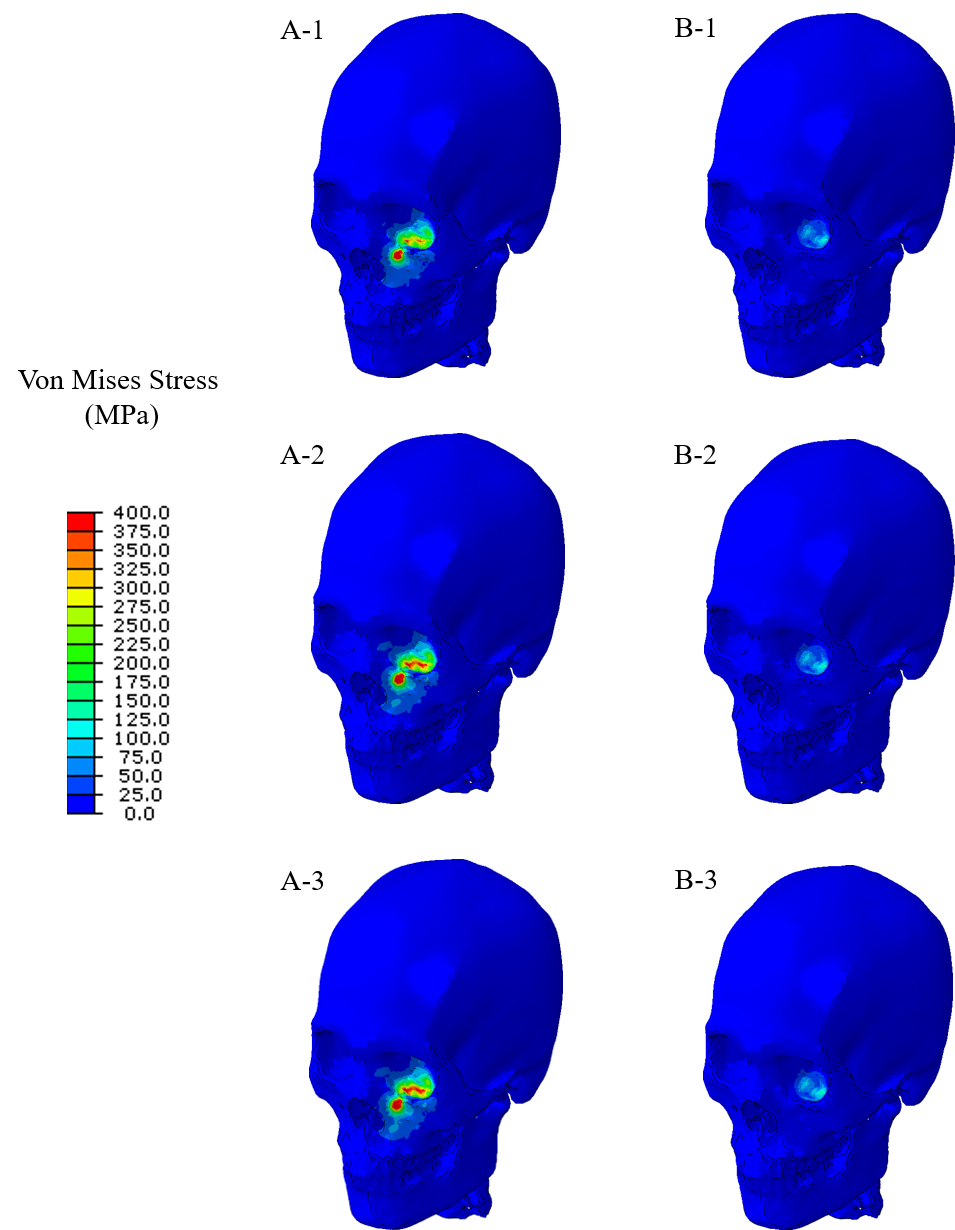

Supplement: Supplementary file 4 [file Image1.TIF]
